# Immersive Video Modeling Versus Traditional Video Modeling for Teaching Central Venous Catheter Insertion to Medical Residents

**DOI:** 10.7759/cureus.13661

**Published:** 2021-03-02

**Authors:** Evan Mah, Julie Yu, Megan Deck, Kish Lyster, Joann Kawchuk, Alison Turnquist, Brent Thoma

**Affiliations:** 1 Department of Family Medicine, University of British Columbia, Campbell River, CAN; 2 College of Medicine, University of Saskatchewan, Saskatoon, CAN; 3 Department of Anesthesiology, Perioperative Medicine, and Pain Management, University of Saskatchewan, Saskatoon, CAN; 4 Department of Anesthesiology, University of Saskatchewan, Saskatoon, CAN; 5 Department of Family Medicine, University of Saskatchewan, Regina, CAN; 6 Department of Emergency Medicine, University of Saskatchewan, Saskatoon, CAN

**Keywords:** medical resident education, video modeling, central venous catheter, skills and simulation training, simulation in medical education, simulation design, video-based learning, 360-degree video recording

## Abstract

Background

Central Venous Catheter (CVC) placement is a common critical care procedure. Simulated practice has been shown to reduce its iatrogenic complications*. *Video modeling (VM) is an instructional adjunct that improves the quality and success of CVC insertion. Immersive VM can improve recall and skill translation, but its role in teaching medical procedures is not established.

Research question/hypothesis

We hypothesized that, relative to traditional VM, immersive VM would decrease cognitive load and enhance ultrasound-guided CVC insertion skill acquisition.

Methods

Thirty-two resident physicians from four specialties were randomized into traditional (control) or immersive VM (intervention) groups for three CVC training sessions. Cognitive load was quantified via NASA Task Load Index (TLX). Mean (± standard deviations) values were compared using two-tailed t-tests. Skill acquisition was quantified by procedural time and the average 5-point [EM1] [TB2] entrustment score of three expert raters.

Results

Overall entrustment scores improved from the first (3.44±0.98) to the third (4.06±1.23; p<0.002) session but were not significantly different between the control and intervention groups. There were no significant differences between NASA TLX scores or procedural time.

Conclusion

We found no significant difference in entrustment, cognitive load, or procedural time. Immersive VM was not found to be superior to traditional VM for teaching CVC insertion.

## Introduction

Central venous catheter (CVC) placement is a medical procedure that involves the insertion of a catheter into the venous system to facilitate the administration of medications, fluids, and blood products [1]. It has been associated with serious mechanical (pneumothorax, bleeding, thrombus formation, occlusion, extravasation, catheter embolism or breakage, fistula formation, air embolism, pericardial tamponade, cardiac aneurysm, or vein stenosis) and infectious (cellulitis, phlebitis, intracardiac abscess, or sepsis) complications. These complications vary in prevalence due to different definitions, reporting patterns, site selection, the catheter used, dressing standards, patient choice, and provider experience [2].

Expert video modeling is a common component of medical simulation training that originated in athletics training [3]. It allows trainees to witness the performance of a procedure to develop self-efficacy and confidence [4]. CVC insertion training that utilizes both video modeling (VM) and task trainers for procedural simulation has been shown to decrease instructor time [5,6], training time [5], equipment spoilage [6], and adverse events [5,7]. The merit of traditional VM has been demonstrated in procedural skills training, particularly in sports [3,8,9]. Recently, more immersive educational technologies, such as virtual reality headsets, have been made available at a reasonable cost, but their application has not been explored in this context.

Immersive VM uses video capture technology and a virtual reality headset that surrounds the user with a convincing replica of the same environment that they might expect in a real-life setting [10]. Immersive VM has been demonstrated to promote learner engagement in the tasks they observe [11-14]. This increased engagement could conceivably improve retention and learning. Additionally, immersive VM has a complicated impact on cognitive load. Frederiksen et al. found that performing procedures in immersive virtual reality increases cognitive load [15]. However, cognitive load decreased with repeated exposure. They hypothesized that through repeated exposure to higher cognitive load during training, trainees may be better prepared for complex real-world performance [15].

Building on this literature, we hypothesized that immersive VM would be acceptable to learners and that, relative to traditional VM, immersive VM would result in enhanced ultrasound-guided CVC insertion skill acquisition mediated by the decreased cognitive load during procedure performance.

This research was previously presented at the University of Saskatchewan Medical Education Research and Scholarship Day (June 8, 2018), the College of Medicine Fall Poster Day (November 23, 2018), and the Canadian Conference on Medical Education (April 14, 2019).

## Materials and methods

The University of Saskatchewan Research Ethics Board deemed this study exempt from ethical review by (BIO# 18-46). The Regina Qu’Appelle Health Region provided operational approval.

All 32 first-year residents from four training programs (anesthesia, emergency medicine, general surgery, and internal medicine) in Saskatoon and Regina (Saskatchewan) were enrolled in a CVC insertion training program at our institution and were invited to participate in the study. Consent was provided by each participant prior to the first session and demographic information was collected (Appendix A).

Expert instructional procedural videos were created simultaneously in traditional (Video 1) and immersive (Video 2) video formats. The traditional format was a fixed, two-dimensional video viewed on the screen of a Samsung S6 32GB cell phone (Samsung Electronics, Suwon, South Korea). The immersive format was a video of a 180-degree wide field of view that was viewed on a Samsung S6 32GB cell phone placed in a Samsung Gear Virtual Reality Headset (2016 edition, Samsung Electronics). The headset allowed the user to raise, lower, and turn their head to change their visual focus and prevented visual input from their true surroundings. We recorded the instructional procedural videos using a CVC Insertion Kit (Teleflex, Wayne, USA) and a CVC Internal Internal Jugular Task Trainer (Simulab, Seattle, USA) with a Samsung S6 32GB cell phone (Video 1*)* and a 360fly 4K Video Camera (360fly, Canonsburg, USA) (Video 2). Both videos were edited with the same instructive audio narrative using Premiere Pro (Adobe, Inc., San Jose, USA). Equipment costs are outlined in Appendix B.

**Video 1:** Traditional Video Model

**Video 2:** 360 Video Model v2.0

The ultrasound-guided, CVC insertion training program spanned three three-hour sessions over a 12-week period. Each group contained three to four residents. All residents were provided with prereading material outlining the procedure. At the beginning of the first session, all residents received procedural instruction by a staff physician experienced in CVC insertion. The instructor taught a standardized, step-wise approach to CVC insertion based upon our institution’s best practices. The resident groups were randomized to the control (traditional VM) or the interventional (immersive VM) groups. We aimed to maintain the composition of each instructional group throughout the study, but in cases where this was not possible because the residents changed instructional groups, they conducted their VM consistent with the study group that they were initially assigned. Following the viewing, both groups practiced the skill on task trainers. Individualized feedback was provided concurrently by the instructors throughout the practice session. The subsequent two training sessions followed the same video review and practice format but did not include dedicated instruction at the beginning. Concurrent feedback was provided by instructors through all of the sessions.

At the end of each of the three training sessions, each participant completed a one-on-one testing session supervised by a staff physician instructor. These sessions were video recorded to include the procedural area, the participant’s hands and arms, and the ultrasound screen. No feedback was provided during this test session. After each session, participants were asked to complete a NASA Task Load Index (TLX), a multidimensional scoring system that assesses cognitive workload (Appendix C), while the observing instructor completed an assessment form consisting of the O-Score entrustment scale [16] and narrative feedback. Participants were stopped by the instructor if they exceeded 15-minutes of procedural time. Time zero began with the first needle insertion into the skin and concluded with the placement of the Tegaderm™ (3M Company, Siant Paul, USA) dressing to secure the CVC. Task success was defined as the completion of the task within the time frame. Failure was defined as the failure to complete the procedure within the allotted time.

**Figure 1:**
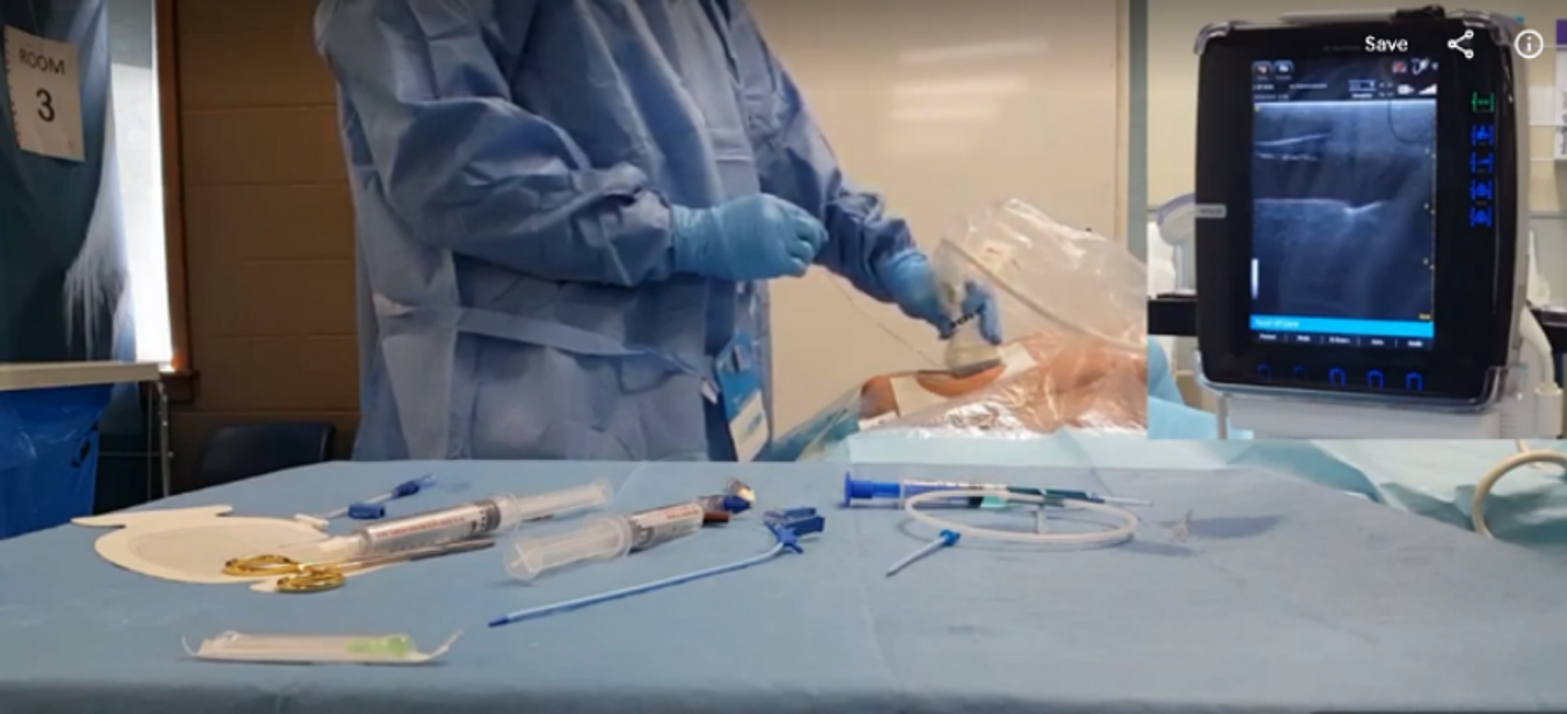
A sample of the video created from a participant’s test session, showing their performance with the video of the ultrasound matched in a picture-in-picture view

Following each participants’ testing session, their procedural and ultrasound videos were combined into a single video (Figure 1). These videos were saved on a password-protected hard drive with file names based on a computerized random number order generator from 100-400. Time from first needle insertion to Tegaderm™ placement was determined by a blinded study investigator. Two additional staff physician investigators, who were not involved in the participant’s recorded teaching session but were familiar with the curriculum and its assessment, performed blinded assessments of each recorded CVC insertion using the O-Score entrustment scale completed via SurveyMonkey (SVML Inc., San Mateo, USA) (Appendix D).

Procedural competence was assessed using the average of the three entrustment scores (one bedside rater and two video raters) and procedure length (from first needle insertion to Tegaderm™ placement). Cognitive load during simulated CVC placement was assessed using the NASA TLX score completed by each participant following each test session. These variables were compared between sessions within groups, as well as between groups using paired and unpaired two-tailed t-tests as appropriate. A power calculation conducted using an alpha of 0.05 and a beta of 0.8 seeking a one-point difference on the entrustment score between the groups required two groups of 16 participants.

Lastly, the acceptability of immersive and traditional VM was explored by comparing CVC insertion training program evaluations from residents in each group. The program was evaluated using a modified version of the evaluation of technology-enhanced learning materials learner perspective (ETELM-LP) survey [17]. Participants completed the survey online using SurveyMonkey (Appendix E) after all three sessions were completed and the results were compared by group using t-tests.

## Results

All 32 first-year eligible for the study participated. Five additional third-year residents participated in the training program but were not eligible for the study. Group demographics are outlined in Table 1. All study participants were right-handed. Most participants (n=19 or 59.4%) were internal medicine residents. Nine participants (28.1%) had received formal instruction on CVC insertion prior to the training program. The control and intervention groups differed in size because the residents were randomized by group and the number of eligible residents in each group varied. 

**Table 1:** Demographic characteristics of the control and intervention groups

	Male	Female	Previous training	Reviewed course material prior to the first session	
				Yes	Partially
Control	13 (72%)	5 (27.7%)	6 (33%)	10	3
Intervention	10 (71%)	4 (28.6%)	3 (21%)	8	6

**Figure 2:**
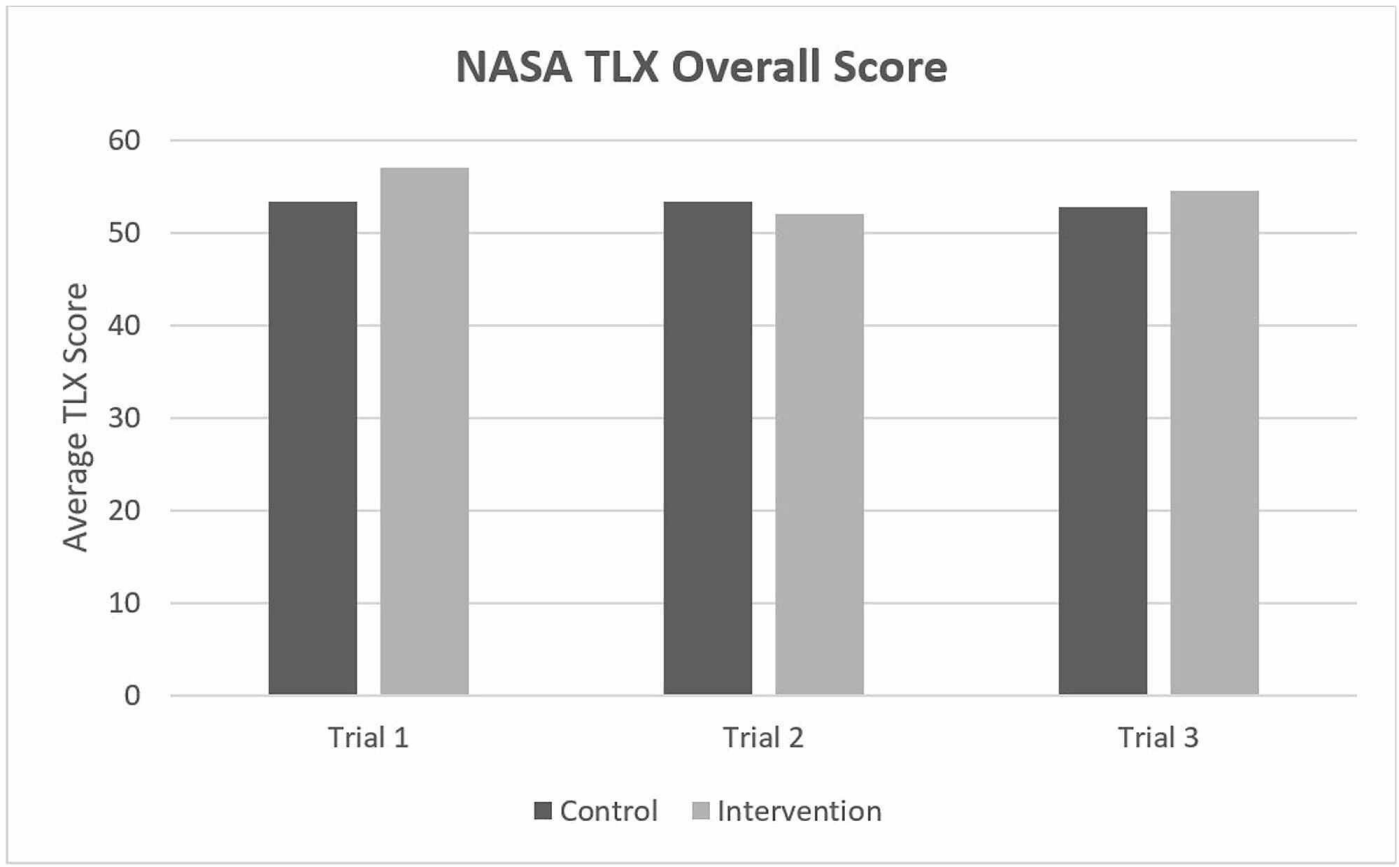
A graph of the average NASA TLX scores for both groups over the three trials. There were no statistically significant differences between groups or between testing sessions. TLX: Task Load Index

**Figure 3:**
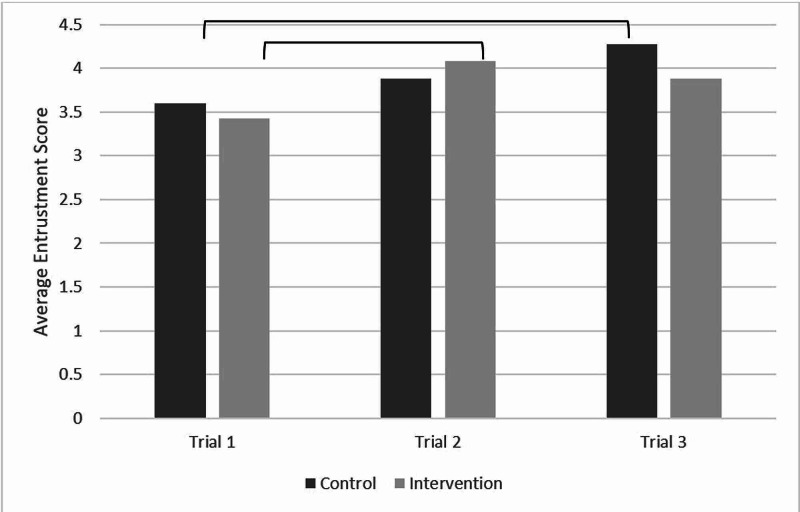
Average entrustment scores for both control and intervention groups over the three testing sessions. The bars indicate there is statistically significant difference at p<0.05.

**Figure 4:**
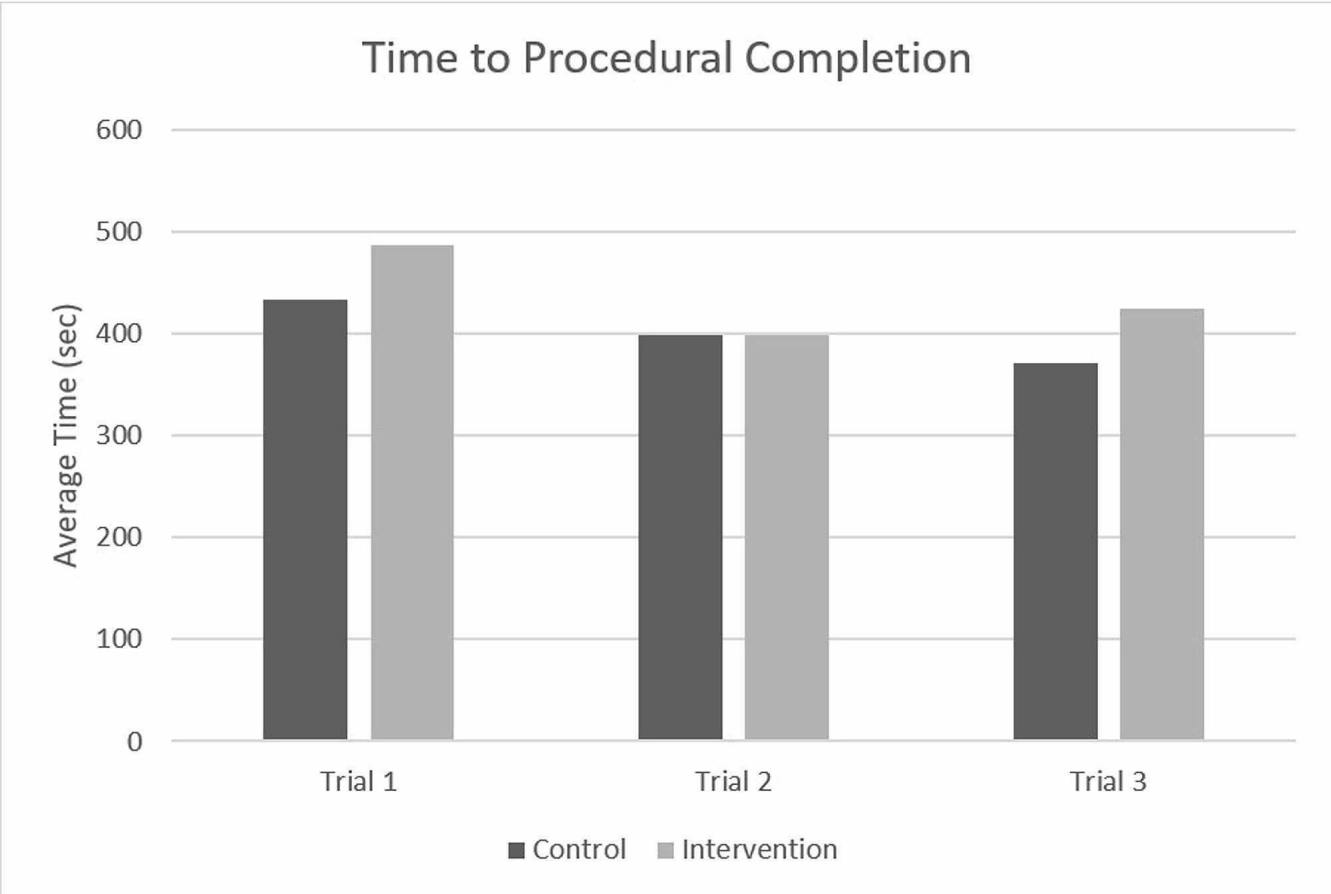
The time to procedural completion for both control and intervention groups over the three testing sessions. There were no statistically significant differences between groups or between testing sessions.

Cognitive load (as assessed by the NASA TLX) and procedural competence (as assessed by procedural time and entrustment score) are reported in Figures 2-4. There was no difference in cognitive load during the procedure in the immersive VM group. Further, there was no change in cognitive load through the three study sessions. There was a significant improvement in entrustment score (mean±SD) from the first (3.4±1.0) to the third (4.1±1.2; p<0.001) session across all participants. There were no statistical differences in entrustment scores between the control and intervention groups during the last session. Time to procedure completion did not significantly change from the first to the third session in either group.

Each item on the ETELM-LP (Appendix F) was rated from strongly disagree (1) to strongly agree (7). Significant differences between the groups are outlined in Table 2. Participants in the control group reported higher agreement that navigation of the technology-based components of the course was logical, consistent, and efficient; that the technology and media supported the learning objectives; that the course did not require inappropriately high technology skills; and that they did not have significant technical problems during the course. The control group also reported higher agreement that the quality of the video review helped them learn the skill, contributed to the achievement of the learning objectives, and was consistent with the instructor’s teaching of the pre-course material.

**Table 2:** Statistically significant differences from the ETELM Learner Perspective Survey between the control and intervention groups ETELM: evaluation of technology-enhanced learning materials

ETELM Question	Control Average	Intervention Average	p-value
Navigation of the technology-based components of the course was logical, consistent, and efficient	6.3	5.4	0.03
The course technologies and media supported the learning objectives	6.3	5.5	0.05
This course did not require inappropriately high technology skills	6.7	5.8	0.005
I did not have significant technical problems during this course	6.5	5.3	0.02
The quality of the video review of the procedure helped me to learn the skill	6.4	5.5	0.03
The video review of the procedure was consistent with the instructor's teaching and pre-course material	6.4	5.6	0.02
The video review of the procedure contributed to the achievement of course learning objectives	6.5	5.5	0.008

## Discussion

Contrary to our hypotheses, immersive VM did not significantly reduce the procedural cognitive load or improve the competence of first-year resident physicians relative to traditional VM. Further, the results of the student evaluations suggest that traditional VM was preferred by learners.

Despite the strong literature base for the use of VM [3,9,18,19], we are unaware of any previous studies investigating its interplay with cognitive load. Early investigations of immersive video technologies [11-15] suggest that they are acceptable to learners and can be an effective educational tool [20]. Building on recent literature suggesting that immersive VM initially increases procedural cognitive load but that this cognitive load decreases with time [15], we hypothesized that incorporating immersive VM at the beginning of procedural training could decrease cognitive load during successive attempts at the procedure. However, our study suggests that this did not occur between sessions or the two groups. There are multiple potential reasons for this: the testing environment may have been persistently stressful, the introduction of a new VM technology may have resulted in an increased extraneous load that further complicated skill acquisition [21], or the three three-hour sessions may not have provided enough time to develop the complex task schema required for CVL insertion, thereby influencing cognitive load and working memory.

Previous research demonstrated increasing procedural competence over the course of multi-session CVC training programs [19-21]. Our findings of improving entrustment scores from the first to last session reflect this, but we did not find a significant difference between the two groups. There could be several reasons for this. Firstly, the concurrent feedback provided in both sessions allowed for the opportunity to facilitate deliberate practice, a method of teaching that depends upon focused, repetitive practice of skill improvement, with feedback [22,23]. The quality of our in-task, concurrent feedback may have overshadowed any positive effect immersive VM could have had on the scoring. This has been recently studied in novice medical students [24], wherein early procedural learning benefitted from VM, but in later procedural interventions students subjectively benefitted more from concurrent feedback. Secondly, we identified that the cognitive load remained higher in the immersive VM group, which may have adversely affected performance. Thirdly, the benefit of VM in the theoretical model of deliberate practice may be rooted in the learners' ability to observe and analyze the performance of experts at key decision points, with expert guidance [23]. Entrustment scores in simulated environments may be an indicator of competence in the clinical setting [25], suggesting that the improvements seen in this workshop may translate to enhanced patient care [26]. Procedural time is also a recognized surrogate of procedural mastery, with the level of experience being inversely related to time [21,25-27]. However, we did not find this association in our study. This may have related to the heterogeneity of specialty programs participating in the study or the amount of time (four weeks) between the three training sessions.

Comparisons of the course evaluations between the control and intervention group raise additional concerns regarding the viability of immersive VM for procedural training. While support was available during the sessions for the residents, the results demonstrate significantly lower ratings for the immersive VM group. While statistically significant, this may not reflect a clinically relevant significance to learners and requires a more in-depth assessment as to the reasons behind these differences. Nevertheless, this suggests that despite the initial enthusiasm for this technology from learners [11-15], in the time frame and workshop format provided in this study, it may not have achieved the ease of use or familiarity to learners.

There were limitations to our study. First, we intentionally sought to conduct a pragmatic study by utilizing commercially available equipment. While we achieved this objective, it is possible that more advanced recordings and VM could have been incorporated with additional funds and equipment that may have resulted in an improved learner experience. Second, there were user-onboarding difficulties in the use of the commercial virtual reality (VR) headsets for the learners and instructors. Despite being a commercial product designed for recreational use, there were limitations related to internet connection speeds, user-interface challenges, and navigation within the VR software. As learners and instructors became familiar with the hardware, this became less of a barrier. Third, there was a delay in the completion of the NASA TLX questionnaire by a small minority of participants despite the time being provided for this during the session. This may have subjected these results to recall bias. Finally, 28% of the participants had previous formal training with CVC insertion. These were evenly distributed between groups, however, this study did not specifically investigate the extent of this formal training, and thus may have influenced the outcomes of interest.

Our study did not demonstrate the benefit of immersive VM over traditional VM, however, there will continue to be opportunities for further investigation in this field. In the short-term, it may be worth investigating the impact of immersive VM for teaching more complex procedures (e.g., intraoperatively) than ultrasound-guided CVC insertion as it may have a larger benefit in these contexts. In the long-term, the permeation of immersive experiences and technologies may become more commonplace, decreasing their cognitive load. Immersive technologies and augmented reality technologies will continue to rapidly evolve and become more intuitive. It will be important to continue to investigate the use of this and newer technology for medical procedural training as it becomes available.

## Conclusions

Simulation using task trainers and video modeling is an effective way to teach medical procedures associated with iatrogenic complications in a low-risk environment. Increasingly sophisticated forms of video modeling have recently become readily available. We hypothesized that teaching ultrasound-guided CVC insertion using immersive VM technology would provide benefits over traditional video modeling. However, the use of immersive VM did not change the cognitive load or improve outcomes over traditional VM when used to augment simulation training for novice resident physicians learning this procedure.

## Figures and Tables

**Figure 5:**
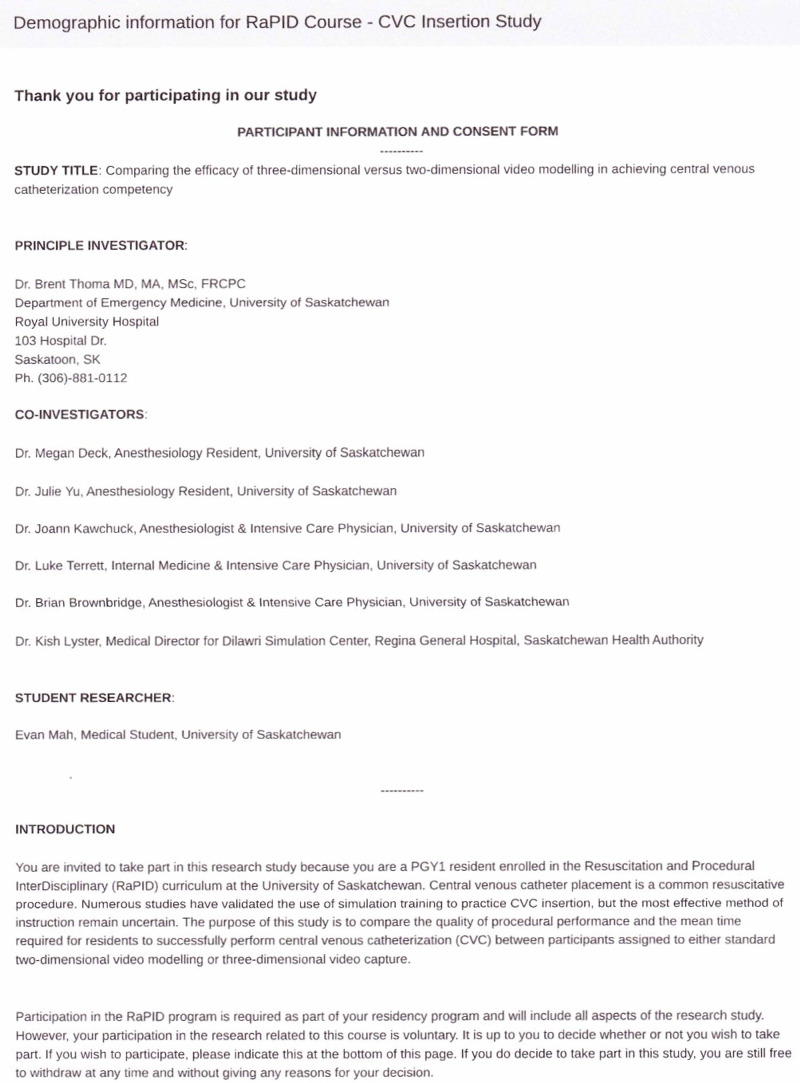
Appendix A-1

**Figure 6:**
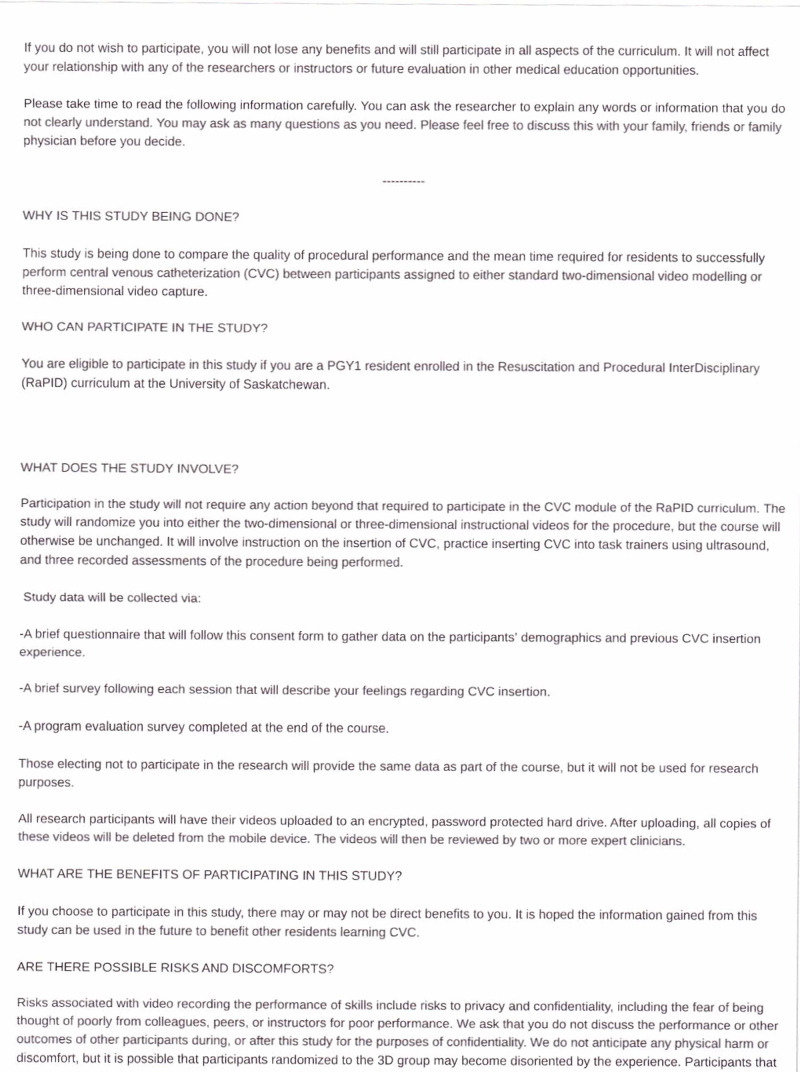
Appendix A - 2

**Figure 7:**
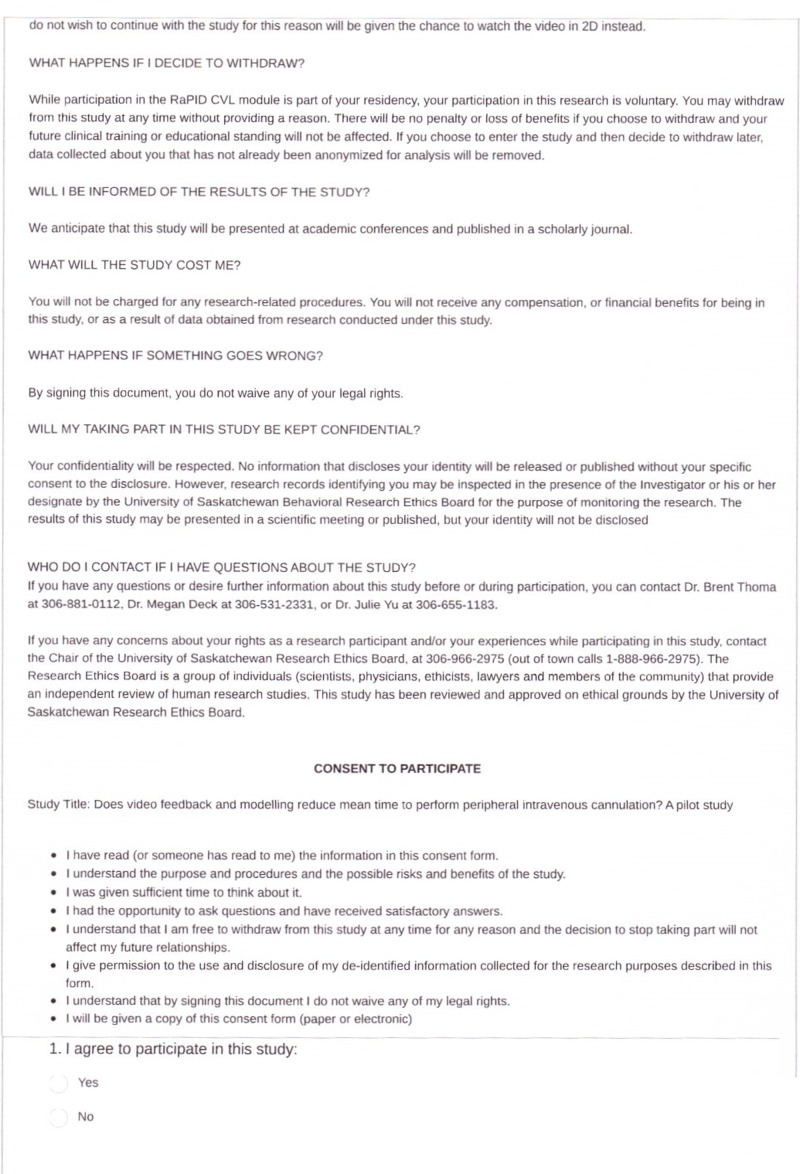
Appendix A - 3

**Figure 8:**
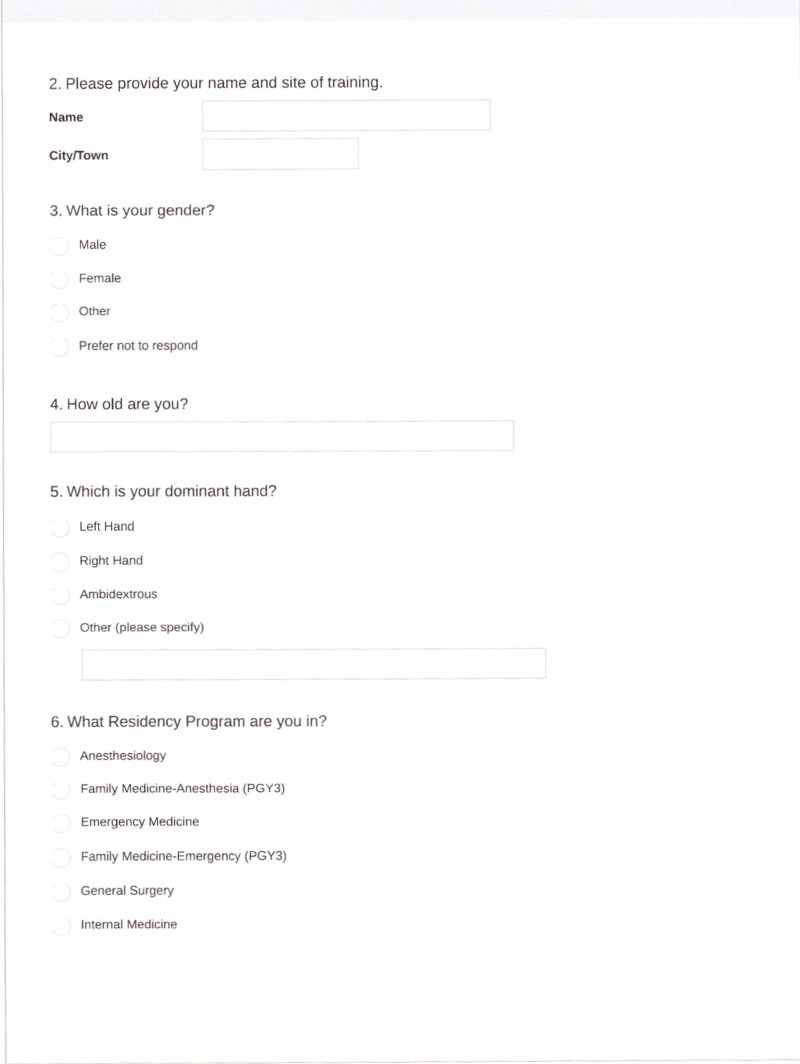
Appendix A - 4

**Figure 9:**
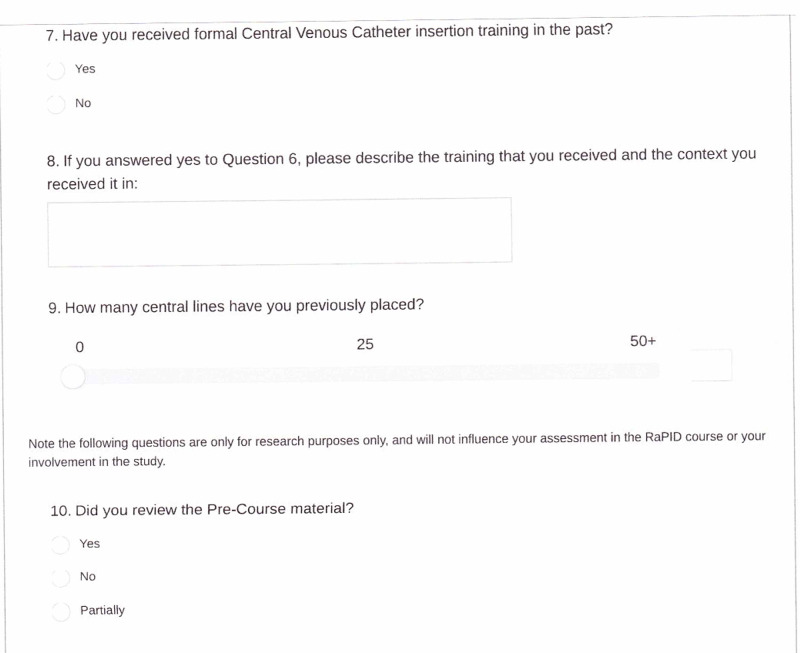
Appendix A - 5

**Figure 10:**
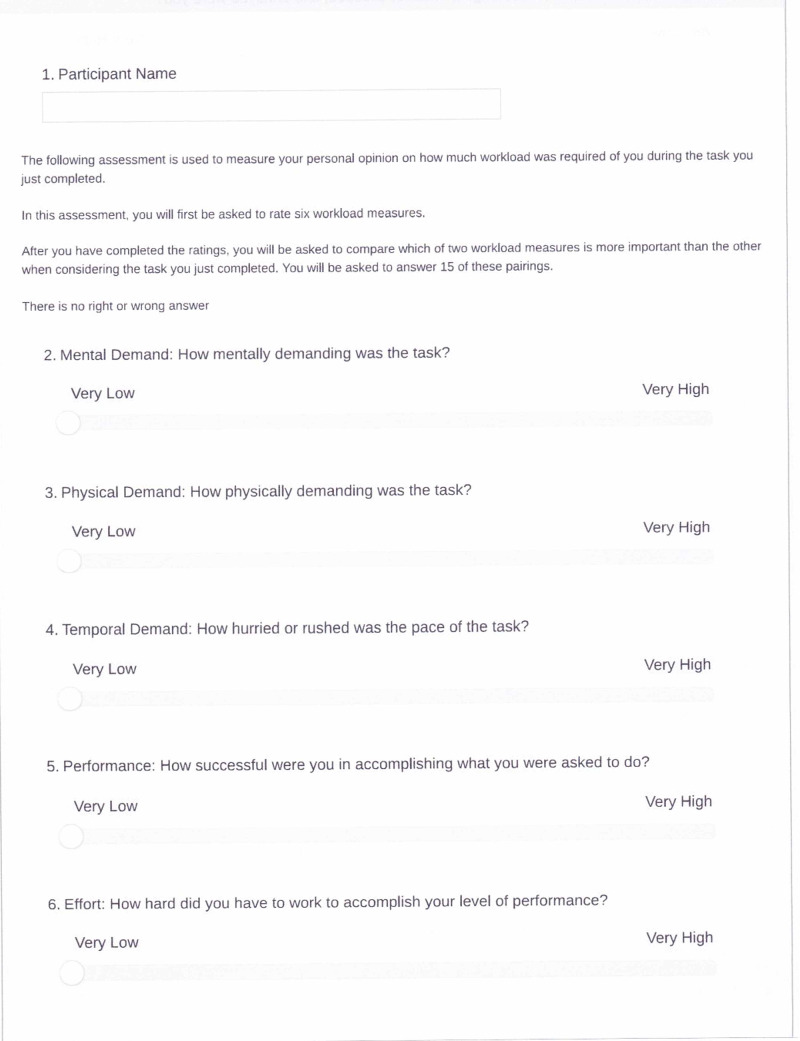
Appendix C - 1

**Figure 11:**
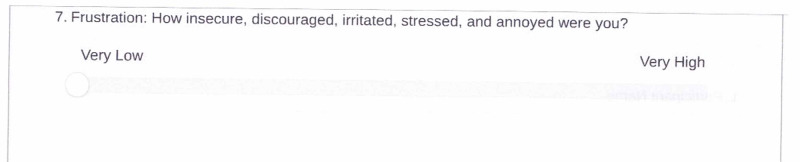
Appendix C - 2

**Figure 12:**
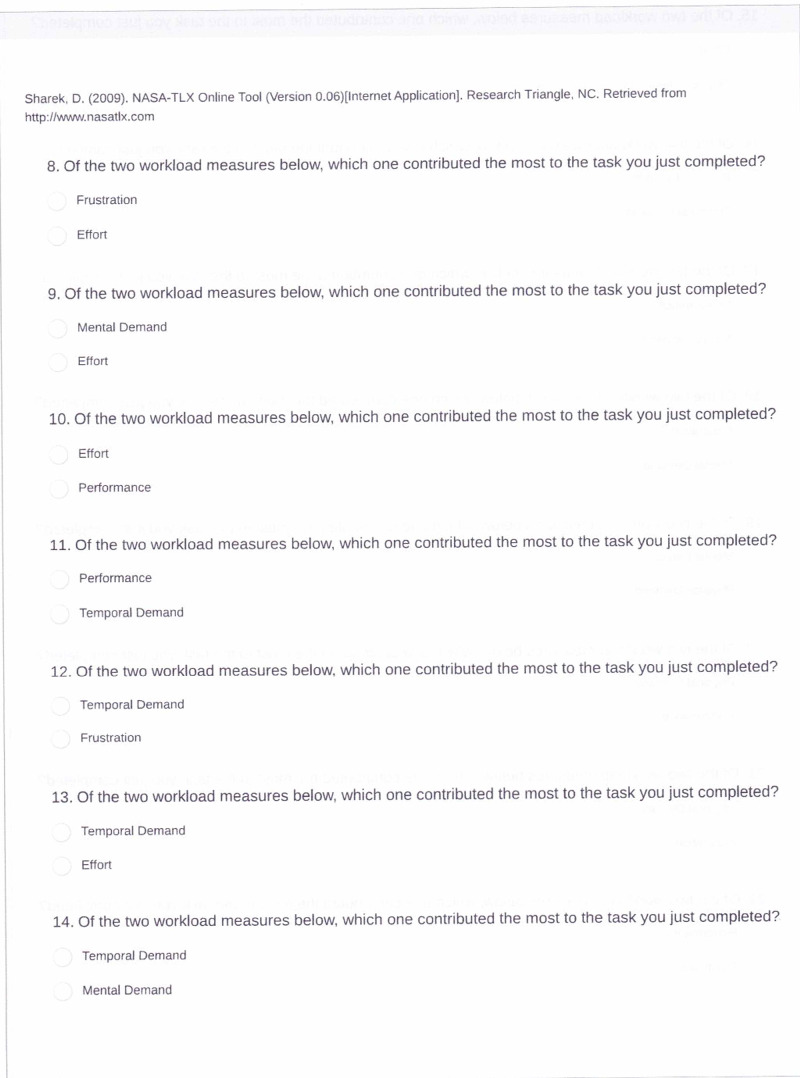
Appendix C - 3

**Figure 13:**
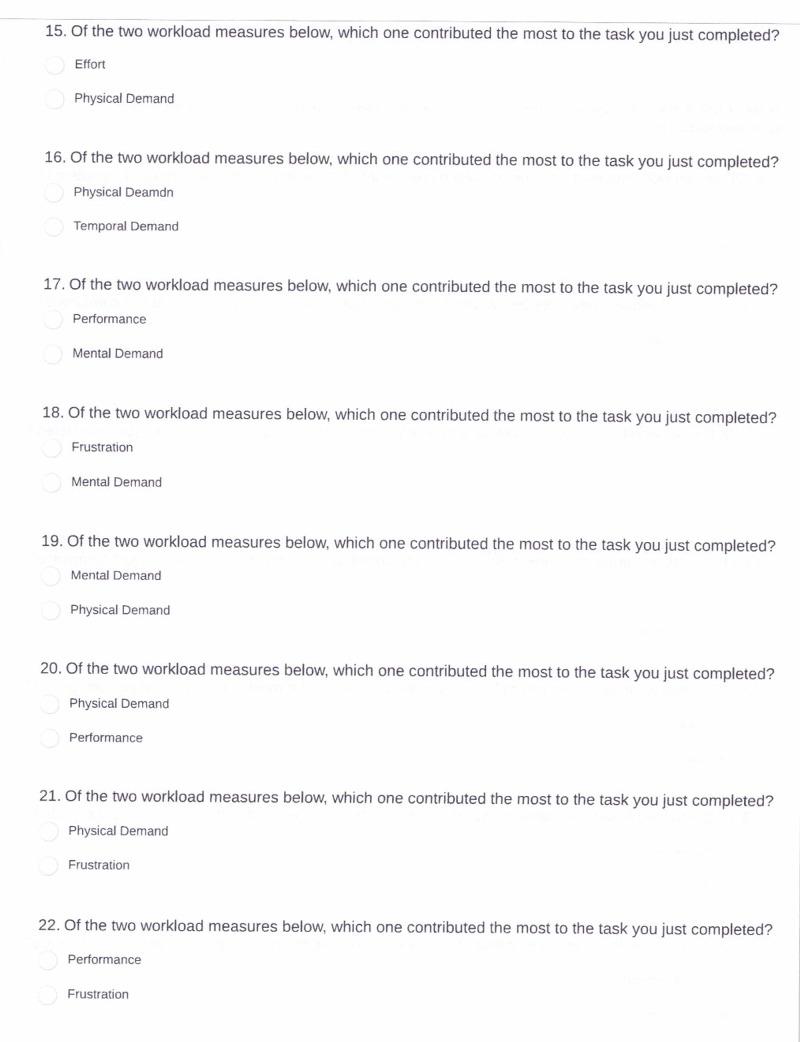
Appendix C - 4

**Figure 14:**
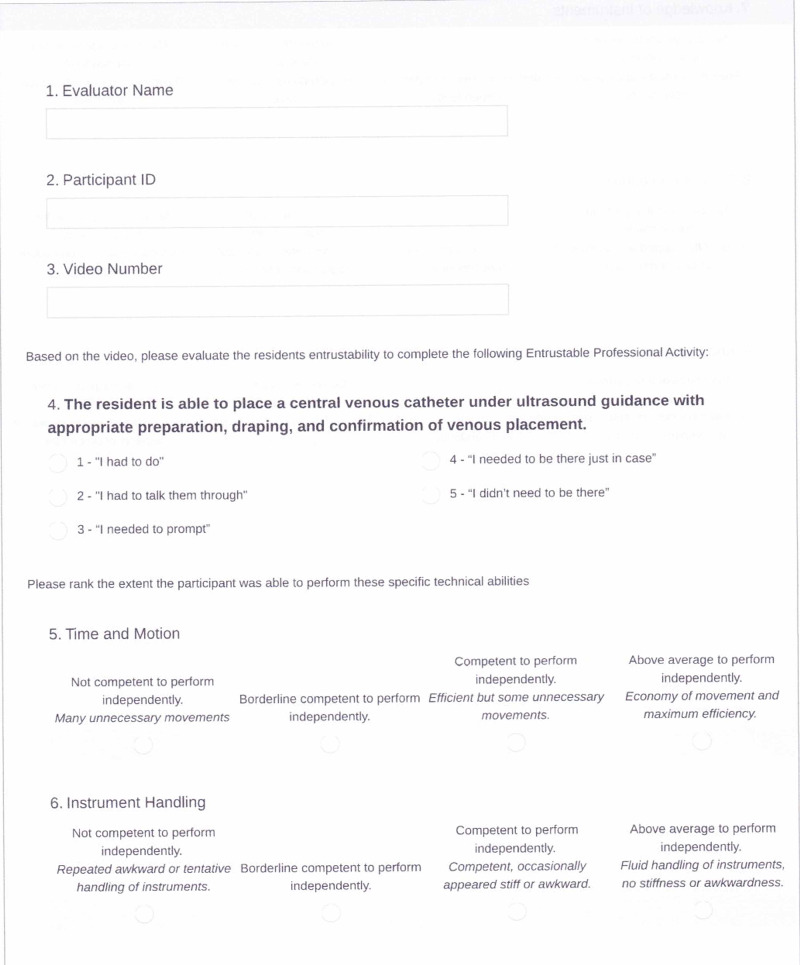
Appendix D - 1

**Figure 15:**
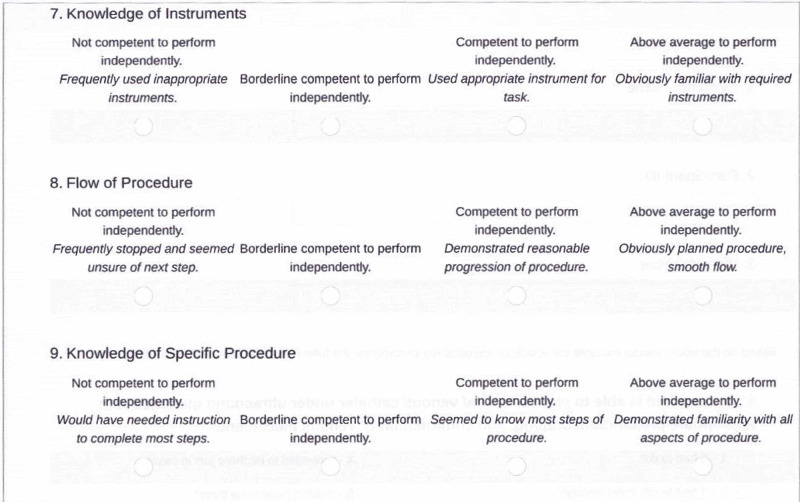
Appendix D - 2

**Figure 16:**
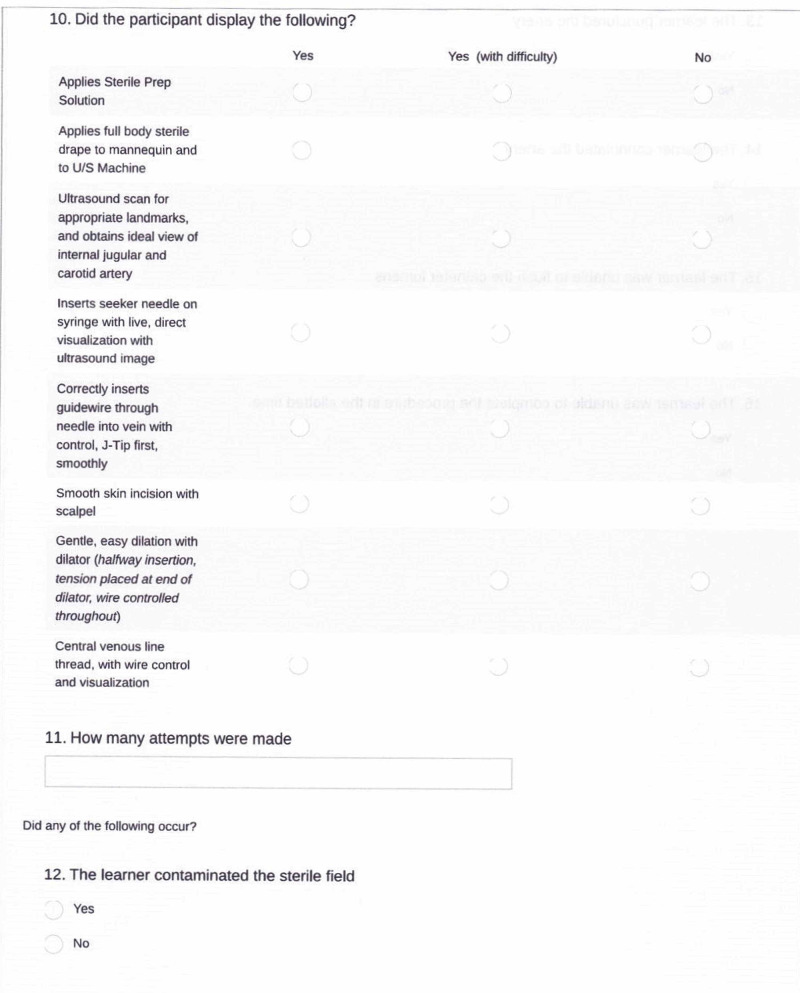
Appendix D - 3

**Figure 17:**
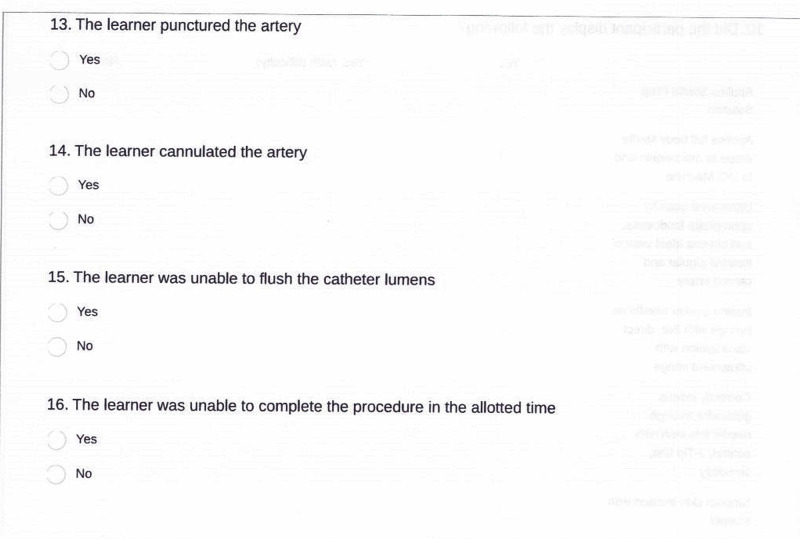
Appendix D - 4

**Figure 18:**
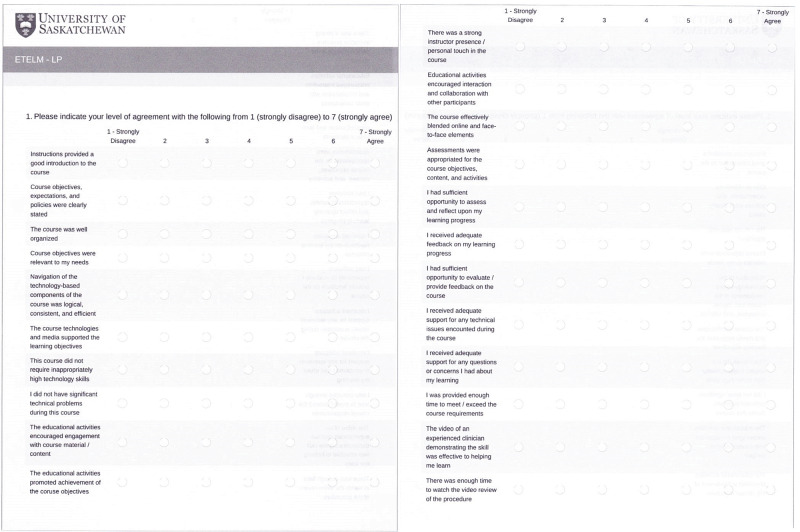
Appendix E - 1

**Figure 19:**
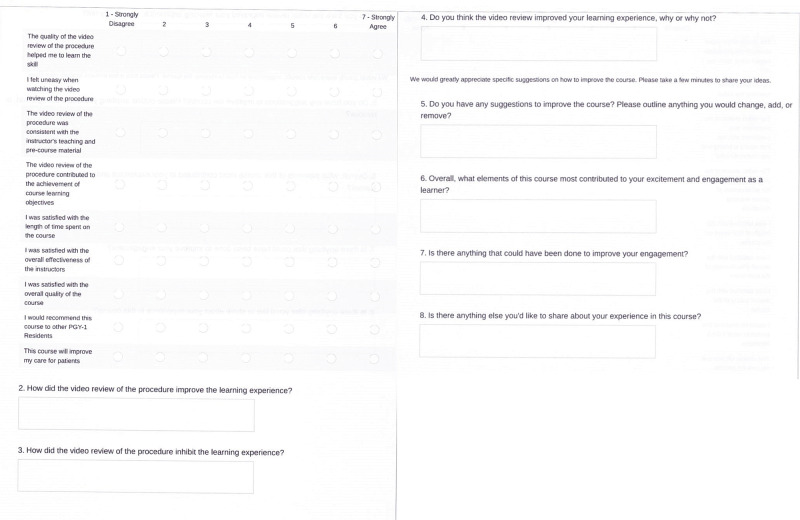
Appendix E - 2

## Appendices

Appendix A

Appendix B

**Table 3:** Itemized list of utilized program equipment

Item	Vender	Quantity	Total Cost (CAD)
360fly 4K Camera	Amazon	1	$469.96
Samsung S6 Smart Phone	Amazon	4	$2,152.76
3D Virtual Reality Headset (Samsung Gear VR Reality Headset 2016 Edition)	Amazon	4	$259.96
360 Camera Mount	Amazon	1	$168.99
External Hard Drive (Seagate Backup Plus Slim 1TB)	Amazon	1	$79.96
Samsung S6 Camera Mount	Amazon	4	$107.20
Adobe Video Editing Software (Adobe Premiere Pro)	Adobe	1	$239.98 (USD)
CVC Insertion Kits	Teleflex	151	$10,570
CVC Femoral Task Trainers	Simulab	2	$6,600
CVC Jugular Task Trainers	Simulab	2	$5,400
CVC Femoral Task Trainer Inserts	Simulab	6	$10,200
CVC Jugular Task Trainer Inserts	Simulab	6	$7,800

Appendix C

Appendix D

Appendix E

Appendix F

**Table 4:** Modified evaluation of technology-enhanced learning materials - learner perspective (ETELM-LP) results 1: Strongly Disagree; 2: Disagree; 3: Somewhat Disagree; 4: Neither Agree Nor Disagree; 5: Somewhat Agree; 6: Agree; 7: Strongly Agree

ETELM Descriptor	Average Rating
Instructions provided a good introduction to the course	5.9
Course objectives, expectations, and policies were clearly stated	6.2
The course was well organized	5.9
Course objectives were relevant to my needs	6.2
Navigation of the technology-based components of the course was logical, consistent, and efficient	5.8
The course technologies and media supported the learning objectives	5.9
This course did not require inappropriately high technology skills	6.2
I did not have significant technical problems during this course	5.9
The educational activities encouraged engagement with course material / content	6.0
The educational activities promoted achievement of the course objectives	6.1
There was a strong instructor presence / personal touch in the course	6.2
Educational activities encouraged interaction and collaboration with other participants	5.8
The course effectively blended online and face-to-face elements	5.7
Assessments were appropriate for the course objectives, content, and activities	6.0
I had sufficient opportunity to assess and reflect upon my learning progress	6.1
I received adequate feedback on my learning progress	6.2
I had sufficient opportunity to evaluate / provide feedback on the course	6.1
I received adequate support for any technical issues encountered during the course	6.2
I received adequate support for any questions or concerns I had about my learning	6.2
I was provided enough time to meet / exceed the course requirements	6.3
The video of an experienced clinician demonstrating the skill was effective to helping me learn	5.9
There was enough time to watch the video review of the procedure	6.1
The quality of the video review of the procedure helped me to learn the skill	5.9
I felt uneasy when watching the video review of the procedure	2.8
The video review of the procedure was consistent with the instructor's teaching and pre-course material	5.9
The video review of the procedure contributed to the achievement of course learning objectives	5.9
I was satisfied with the length of time spent on the course	5.8
I was satisfied with the overall effectiveness of the instructors	6.4
I was satisfied with the overall quality of the course	6.3
I would recommend this course to other PGY-1 residents	6.4
This course will improve my care for patients	6.3
